# Dendritic Cells under Hypoxia: How Oxygen Shortage Affects the Linkage between Innate and Adaptive Immunity

**DOI:** 10.1155/2016/5134329

**Published:** 2016-02-04

**Authors:** Sandra Winning, Joachim Fandrey

**Affiliations:** Institut für Physiologie, Universität Duisburg-Essen, Hufelandstrasse 55, 45122 Essen, Germany

## Abstract

Dendritic cells (DCs) are considered as one of the main regulators of immune responses. They collect antigens, process them, and present typical antigenic structures to lymphocytes, thereby inducing an adaptive immune response. All these processes take place under conditions of oxygen shortage (hypoxia) which is often not considered in experimental settings. This review highlights how deeply hypoxia modulates human as well as mouse immature and mature dendritic cell functions. It tries to link* in vitro* results to actual* in vivo* studies and outlines how hypoxia-mediated shaping of dendritic cells affects the activation of (innate) immunity.

## 1. Introduction

Dendritic cells (DCs) are major directors of immune responses [1]. DCs are part of the innate immune system and function as sentinels for pathogens at potential sites of invasion (e.g., the skin or the gastrointestinal tract). Once they have recognized pathogens DCs capture them and process the respective antigen structures. Proteins are then converted into peptides which are subsequently presented on major histocompatibility complex (MHC) molecules and recognized by T lymphocytes [2]. The dendritic cell drives the immune response depending on the kind of antigen it has taken up. If the host needs defense against an invaded pathogen, DCs present the antigenic peptides to cytotoxic CD8^+^ T cells and proinflammatory CD4^+^ T helper (Th1) cells activating the lymphocytes via direct cell-cell contact and proinflammatory cytokines such as interleukin- (IL-) 12 or IL-6 [3, 4]. If autoimmunity or abundant inflammation needs to be dampened DCs interact with regulatory T cells (Treg) directly and via secretion of anti-inflammatory cytokines such as IL-10 or retinoic acid (RA) [5–7]. What is often hardly considered in experimental settings is the fact that all processes directing the immune response of our organism take place under deprivation of nutrients and oxygen. The interplay of dendritic cells and lymphocytes either takes place in severely inflamed tissue or in secondary lymphoid organs. These tissues have been described to exhibit low oxygen tensions. Oxygen distribution in the spleen and lymph nodes is highly variable and hypoxic lymphocytes have been identified in both organs [8]. The adaptation of cells to reduced oxygen tensions is largely coordinated by hypoxia-inducible factors (HIFs) which have come into the focus of immunological research during the last ten years.

## 2. Criteria for This Review

Inflammatory hypoxia is increasingly recognized as a critical determinant for the immune response. This review focuses on mouse and human dendritic cells and their maturation and activation under hypoxic conditions. By covering publications on* in vitro* studies a particular emphasis is put on HIFs as coordinators of the genetic response to hypoxia. The availability of mice with a DC-specific HIF-1*α* k.-o. now allows a first appreciation of the potential role of hypoxia in DC function* in vivo*.

### 2.1. Hypoxia-Inducible Factors

#### 2.1.1. Posttranslational Modifications

Hypoxia-inducible factors belong to the family of basic helix-loop-helix proteins [9]. They consist of one *α*- (HIF-1*α*, HIF-2*α*, or HIF-3*α*) and one common *β*-subunit (HIF-1*β* or ARNT, aryl carbon nuclear translocator) forming the DNA-binding transcription factor dimer. Whereas the HIF-1*β* protein is not affected by changes in oxygen tension HIF-*α* proteins are only detectable under hypoxic conditions. HIF-1*α* mRNA is expressed and transcribed in all nucleated cells whereas the expression of HIF-2*α* is more limited. HIF-2*α* is found especially in endothelial cells but also in immune cells such as macrophages or dendritic cells. The role of HIF-3*α* remains poorly defined so far, particularly in immune cells, and this review will therefore focus on HIF-1*α* and HIF-2*α*. HIF-*α* proteins are under tight posttranslational control by oxygen. Under normoxia, specific prolyl hydroxylase domain containing enzymes (PHD 1, 2, and 3) use molecular oxygen to immediately hydroxylate distinct prolyl residues of newly synthesized HIF-*α*-subunits. Hydroxylated HIF-*α* (OH-HIF-*α*) interacts with the von-Hippel-Lindau protein (pVHL) E3 ligase complex that polyubiquinates OH-HIF-*α* leading to instantaneous proteasomal degradation under normoxia [10]. Thus, HIF-*α* proteins are oxygen-labile. In contrast, under hypoxia, prolyl hydroxylases lack oxygen as a crucial cosubstrate and are reduced in their enzymatic activity. HIF-*α*s can accumulate, translocate into the nucleus, and dimerize with HIF-1*β*. The transcription factor complexes HIF-1 (HIF-1*α*/HIF-1*β*) and HIF-2 (HIF-2*α*/HIF-1*β*) recruit cofactors such as p300/CBP (cAMP-response element binding protein) and bind to hypoxia-responsive elements of target gene DNA. In addition to proline hydroxylation, HIF-1*α* and HIF-2*α* can be hydroxylated at an asparagine residue in their C-terminal part. Asparagine hydroxylation is controlled by an oxygen-sensitive asparagyl hydroxylase, termed factor-inhibiting HIF- (FIH) 1. FIH-1 activity under normoxia prevents cofactor recruitment and transcriptional activity of the HIF complex. HIFs have been shown to regulate more than 100 genes that are involved in glucose metabolism, cell death, cell cycle, angiogenesis, and erythropoiesis [11].

#### 2.1.2. Inflammatory Stimulation

During the last decade it has been recognized that many other factors are able to induce HIF-*α* although HIF-1*α* has been studied more thoroughly than HIF-2*α*. Bacterial lipopolysaccharides (LPS) are able to induce the NF-*κ*B pathway and HIF-1*α* mRNA has been shown to be a target of classical NF-*κ*B activation by several groups [12–15]. Recently it was shown that HIF-1 is one of the essential modulators of the cytokine response to bacterial LPS as it is crucial for the synthesis of IL-1*β* [16]. LPS induce intracellular succinate which stabilizes HIF-*α* protein via PHD inhibition. In macrophages, this led to increased IL-1*β* synthesis and release [16]. Furthermore, HIF-1*α* protein and mRNA expression have been shown to be induced in macrophages purified from wounds. The authors could show that elevated protein levels hereby depended on the inflammatory cytokine TNF-*α* [17]. Reactive oxygen species (ROS) have been shown to induce HIF-1*α* as well, although this effect seemed to be time-dependent as external H_2_O_2_ induced HIF-1*α* protein in human osteosarcoma cells at early time points but suppressed it later on. HIF-1 target gene expression was suppressed over the whole time period [18]. Nitric oxide (NO^∙^) has been shown to induce HIF-1*α* in normoxic macrophages stimulating macrophage migration by modulating the actin cytoskeleton via small GTPases [19]. The role of HIF-2 in the function of immune cells has been studied much less. Imtiyaz et al. [20] have characterized macrophages lacking HIF-2*α* under immunological settings. Macrophage NO^∙^ production and expression of costimulatory molecules CD86 and MHCII were unaffected by loss of HIF-2*α*, but Imtiyaz and coworkers found profound changes in cytokine mRNA expression and protein release after stimulation with LPS and interferon *γ* (IFN*γ*). Murine bone-marrow-derived macrophages lacking HIF-2*α* showed reduced induction of IL-1*β*, IL12p35, Cxcl2, and IL-6 mRNA levels under inflammatory hypoxia whereas hypoxia alone if at all only moderately affected mRNA expression of these genes. Moving to* in vivo* models the authors found that macrophage HIF-2*α* was required for an adequate immune response to cutaneous and peritoneal irritants [20]. Furthermore, loss of macrophage HIF-2*α* prevented infiltration of tumor associated macrophages (TAMs) in models of hepatocellular carcinoma and colitis-associated cancer which inhibited tumor growth [20]. The role of HIF-2 in the function of dendritic cells under hypoxia is almost completely unexplored, although Filippi et al. could show that human dendritic cells express HIF-2*α* mRNA [21].

HIF-1 activity has been shown to be induced by a number of viral infections as well. The human immunodeficiency virus HIV-1 induces HIF-1*α* protein via induction of intracellular ROS. HIF-1 associates with the HIV long-term repeat to induce HIV gene transcription [22]. Hepatitis Bx protein (Hbx) increases the stability of HIF-1*α* protein via p42/44 mitogen-activated protein kinases (MAPK). Transactivation of HIF-1 is also increased as Hbx induces the interaction of the transcription factor with CBP, one of the cofactors HIFs recruit for DNA binding [23]. Kaposi's sarcoma-associated herpesvirus (KSHV) and Epstein-Barr-Virus (EBV) induce HIF-1*α* protein by increasing the proteasomal degradation of proteins involved in the normoxic degradation of HIF-1*α*. LANA, KSHV latency-associated nuclear antigen [24], increases the degradation of pVHL whereas the PHDs 1 and 3 are degraded by proteasomes upon EBV infection [25]. Furthermore, KSHV expresses functional HREs in the promoter regions of viral genes which could be activated* in vitro* by binding of either HIF-1*α* or HIF-2*α* [26]. Taking these data into consideration, activation of HIF-1 (and potentially HIF-2) in viral infections seems to bring benefit to the pathogen rather than the host.

### 2.2. Dendritic Cells and HIFs

DCs are central in coordinating immune responses against pathogens: whenever they register pathogens they pick them up, process the proteins of pathogens, and present typical antigenic peptides to cells of the adaptive immunity. These processes do not only activate the cell but also induce DC differentiation and maturation and make them migrate towards secondary lymphoid organs. DC differentiation and maturation are accompanied and at least in part defined by the upregulation of costimulatory molecules such as CD80 and CD86 [27]. Furthermore, mature dendritic cells express high levels of surface MHCII and CD40 and are able to secrete IL-12 [27]. This is important as DCs have to function as antigen presenters and producers of IL-12 at the same time to induce differentiation and proliferation of Th1 cells [3]. Until now, several groups have analyzed DC function under hypoxic conditions.

### 2.3. *In Vitro* Analysis

For human DCs it has been shown that differentiation of DCs from blood monocytes under hypoxia resulted in a more active phenotype exhibiting a higher ability to stimulate allogeneic T cell responses. Furthermore, hypoxic immature DCs have been shown to downregulate bacterial phagocytosis and exhibit an increased migratory capacity. Several groups have contributed to the current knowledge on the underlying mechanisms: Elia et al. [28] and Ricciardi et al. [29] have found higher expression of the costimulatory molecules CD80, HLA class II, and CD86 on the cellular surface of hypoxic immature DCs. In contrast, costimulatory molecule expression appeared not to be different between mature hypoxic or normoxic DCs [28]. Ogino et al. [30] reported that hypoxic immature like mature DC exhibited higher allo-T cell stimulation than normoxic DCs. This finding was partly supported by Elia et al. [28] who also found this effect for hypoxic immature DCs. Spirig et al. [31] exposed immature monocyte-derived DCs to hypoxia but could not detect any differences in the expression of costimulatory markers. In turn, LPS-mediated maturation of these cells was augmented by hypoxic conditions [31]. Rama et al. [32] in contrast observed a hypoxia-mediated differentiation of immature human monocyte-derived DCs. They exposed the cells for longer time periods to harsher hypoxic conditions than Spirig and coworkers and could detect a higher expression of CD40 after hypoxic treatment. Hypoxia also led to higher T cell stimulatory activity of DCs and this effect could be blocked by inhibition of the HIF pathway [32]. Of note, maturation of DCs under hypoxia leads to a shift in the expression of chemokines and chemokine receptors. While chemokines get downregulated chemokine receptors are upregulated indicating that hypoxia may favor migratory capacity of mature DCs carrying cytokine receptors rather than immunologic functions such as immune cell recruitment [29, 33]. Filippi et al. could recently show that short-term hypoxia induces the migratory capacity of immature and mature DCs in an* in vitro* migration assay and that this effect depends on the expression of HIF-1*α* [21]. In contrast to the enhanced migratory capacity hypoxic DCs exhibit a reduced phagocytic activity. Several groups have shown that hypoxia downregulated antigen uptake by immature DCs [28, 30] in a manner that seemed to be independent of HIF-1*α* [30]. These in part divergent reports may in fact be explained by some still existing uncertainties about the actual degree of cellular hypoxia under different experimental conditions. In addition, the duration of hypoxic exposure will affect the DC response as well as continuous versus intermittent hypoxia.

Hypoxia did not change costimulatory molecule expression of mature DCs, but several groups have found other hypoxia-dependent changes in mature DC function. Chronic hypoxia promoted the onset of a highly proinflammatory gene expression profile in mature DCs generated from human monocytes [34]. Hypoxic mature DCs thereby showed induced gene expression of cytokines and chemokines that are known to induce endothelial cell survival, recruitment and adhesion of mononuclear phagocytes, and recruitment and activation of predominantly Th1/Th17 cells [34]. Yang et al. [35] extended this knowledge and showed that hypoxic mature DCs upregulated the expression of A2B adenosine receptor (A2BAR) and thereby predominantly induced Th2 activation. In 2011, Bosco et al. found that chronic hypoxia potently induced the cell surface expression of triggering receptor expressed on myeloid cells- (TREM-) 1 on mature DCs [36]. The authors illustrated a transient induction depending on the severity of hypoxia and they identified an HRE in the promoter region of TREM-1. Silencing of HIF-1*α* decreased TREM-1 protein levels. Additional work of the same group has shown that hypoxia induces TREM-1 expression also in immature DCs [37]. TREM-1 cross-linking has been associated with an induced release of inflammatory cytokines such as TNF-*α*, IL-6, and chemokines such as CXCL8, CCL4, and CCL5. In addition, TREM-1 cross-linking has been shown to induce the release of IL12p70, a cytokine inducing Th1 immune responses. This would mean that hypoxia does not only favor migration of mature DCs but also modulates their inflammatory repertoire to attract other immune cells and to direct T cell activation. Thus, for human dendritic cells it seems appropriate when Bosco and Varesio claim a “dendritic cell reprogramming by hypoxic environment” [38]. Examples of how hypoxia affects differentiation and cytokine response of human dendritic cells can be found in Table 1.

Jantsch et al. reported that murine bone-marrow-derived dendritic cells upregulate CD80 and CD86 after exposure to hypoxia and bacterial LPS in an HIF-1-dependent manner. Consistent with these findings DCs cultivated under hypoxic conditions were less efficient in antigen uptake as they showed a more mature phenotype. In contrast, hypoxic treatment (24 h) alone was not able to induce expression of costimulatory molecules on the surface of BmDCs [41]. These findings appear to be in conflict with the results of Köhler et al. This group used a conditional knockdown of HIF-1*α* in BmDCs (by crossing HIF-1*α* ^+f/+f^ mice with CD11c-cre^wt/tg^ animals) rather than a siRNA approach and they kept BmDCs under normoxic or hypoxic conditions during the whole process of differentiation from bone marrow cells [42]. DCs differentiated for six days under hypoxic conditions showed a marked upregulation of CD80, CD86, and MHCII but none of these molecules showed an HIF-1*α* dependent regulation. These somehow contradictory results may be explained by the different exposure times to hypoxia. The work of Köhler et al. instead revealed changes in cytokine expression of hypoxic DCs which were mainly unaffected by loss of HIF-1*α*. Only secretion of IL-22 seemed to be HIF-1*α* dependent when BmDCs were differentiated under hypoxic conditions. In addition, only DCs expressing functional HIF-1*α* showed an increase of surface CCR7 after hypoxic differentiation. This led to a reduced migration of HIF-1*α* deficient dendritic cells [42]. Further experiments regarding DC stimulation with defined pathogens revealed that bacterial CpGs but not viral poly(I:C) were able to stabilize HIF-1*α* protein [43]. Jantsch et al. concluded that MyD88 is essential to induce inflammatory, HIF-1-dependent gene transcription (MyD88 is not involved in intracellular TLR3 signaling triggered by poly(I:C)). One potential target gene of HIF-1 in DCs cultivated under inflammatory hypoxia (hypoxia + LPS or hypoxia + CpGs, resp.) was inducible nitric oxide synthase. Along that line Wobben et al. [44] reported that LPS as well as CpGs could stimulate HIF-1*α* protein in BmDCs generated from control and conditional HIF-1*α* knockout animals (deletion of the DNA binding domain of HIF-1*α* under the control of the lyz2-cre-promoter; BmDC cultures of these mice show knockout in generated DCs, although circulating dendritic cells do not express lyz2-cre-promoter). Furthermore, BmDCs lacking functional HIF-1*α* protein showed severe deficiencies in the release of type I interferons after LPS stimulation and could not induce a proper T cell activation in an* in vitro* CD8^+^ lymphocyte activation assay [44]. Last but not least HIF-1-induced gene expression has been found to modulate the differentiation of murine plasmacytoid DCs (pDCs). Fms-related tyrosine kinase 3-ligand (flt-3L) induced differentiation of pDCs from murine bone marrow cells was dramatically reduced upon hypoxic cultivation and this effect was not found when pDCs were lacking HIF-1*α*. Inhibitor of DNA binding 2 (id2) was identified as suppressor of differentiation. id2 thereby seemed to be exclusively regulated by HIF-1 as a loss of HIF-2*α* in the respective cells could not hinder suppression of differentiation [45]. Examples on how hypoxia affects the differentiation and cytokine response of murine dendritic cells can be found in Table 2.

### 2.4. *In Vivo* Models

Until now, there are only few published studies that address the role of HIFs in dendritic cells in inflammatory settings. Weigert et al. [45] have analyzed the role of HIFs for plasmacytoid DC differentiation and found that HIF-1*α* limited pDC generation in the bone marrow. In its absence, pDC development was encouraged and numbers of pDC increased. When mice with breast tumors in a PyMT-MMTV model were crossed with HIF-1*α* ^+f/+f^ lyz2-cre mice lacking functional HIF-1*α* in myeloid cells Weigert et al. observed markedly enhanced numbers of pDC within the tumors compared with WT controls [45]. Whether this affects tumor progression and disease outcome still has to be analyzed. Köhler et al. [42] have assayed the ability of BmDCs differentiated under hypoxic conditions to get recruited to secondary lymphoid organs. For this purpose, they generated differentially labeled BmDCs from HIF-1*α* ^+f/+f^ and HIF-1*α* ^+f/+f^ CD11c-cre mice under normoxic and hypoxic conditions and injected equal amounts of WT and HIF-1*α* k.-o. BmDCs into the footpad of mice. Due to the different labels they could distinguish how many WT or knockout DCs had migrated towards the popliteal lymph nodes and found that the hypoxic increase in DC migration was HIF-1 dependent [42]. Three other models have been used to elucidate the influence of dendritic cell HIF expression on their interaction with T cells. First, vaccination efficacy has been addressed and second two models of inflammation were investigated, namely,* Leishmania* infection and dextran sodium sulfate- (DSS-) induced murine colitis [46–48]. Bhandari et al. studied HIF-1*α* ^+f/+f^ Tie2-cre mice showing knockout not only in endothelial but also in hematopoietic cells. They vaccinated HIF-1*α* ^+f/+f^ and HIF-1*α* ^+f/+f^ Tie2-cre mice with a synthetic OVA peptide that specifically induced a CD8^+^ T cell response. Analysis of IFN*γ* release by specific T cells in the spleens eight days after vaccination revealed that dendritic cells lacking HIF-1*α* very insufficiently induced T cell activation compared to WT DCs. In addition to this, HIF-1*α* ^+f/+f^ Tie2-cre mice showed reduced titers of specific OVA antibodies [46].

Very recent studies used mouse models with dendritic HIF-1*α* knockout in inflammatory settings* in vivo.* Hammami et al. [47] analyzed HIF-1*α* ^+f/+f^ and HIF-1*α* ^+f/+f^ CD11c-cre^wt/tg^ animals in* Leishmania* infection. Somehow in contrast to Bhandari et al. [46] they found that ablation of HIF-1*α* in dendritic cells resulted in a more efficient immune response. This comprised increased production of IL-12, induced expansion of CD8^+^ cells, and higher frequency of short-lived effector cells, a specialized CD8^+^ T cell population that can be found in resolving acute infection.* Leishmania* infection led to upregulation of HIF-1*α* in splenocytes. This could be shown to restrict CD8^+^ T cell expansion as hemizygous HIF-1*α* ^+/−^ mice exhibited a significantly higher expansion of CD8^+^ cells. HIF-1*α* expression in dendritic cells in turn exacerbated disease [47].

**Figure 1:**
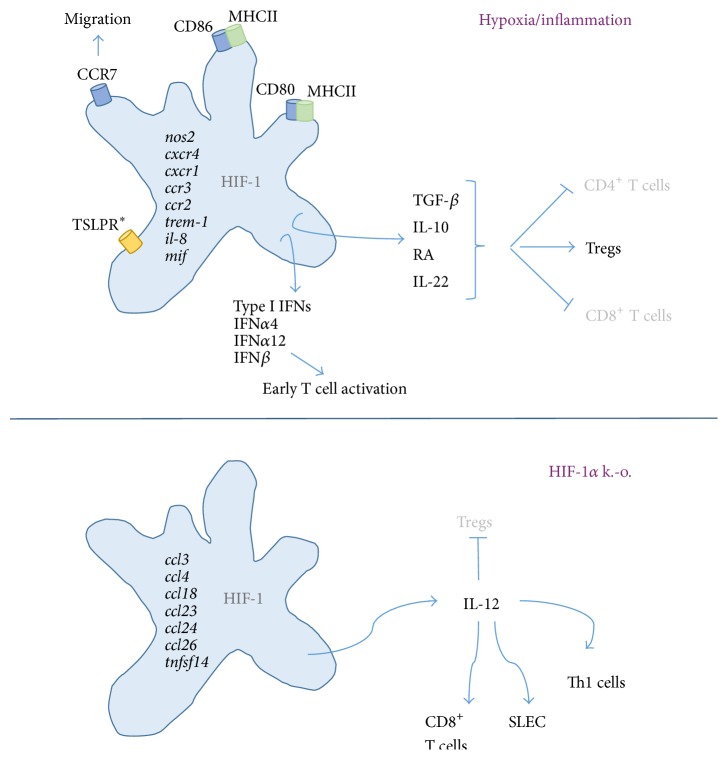
Features of dendritic cells in the presence or absence of active HIF-1*α*. Dendritic cells expressing HIF-1*α* have been shown to induce costimulatory molecules (CD80, CD86, and MHCII/HLA class II) under inflammatory hypoxic conditions. Transcripts of* nod2, cxcr4, cxcr1, ccr3, ccr2, trem-1, il-8*, and* mif* are upregulated. Induced expression of CCR7 favors migration of DC towards secondary lymph nodes. These DCs are potent inducers of Tregs via TGF-*β*, IL-10, RA, and IL-22 although they are able to induce a robust early T cell activation by secretion of type I interferons. Via TSLP, DCs in the gut may be shifted towards a tolerogenic phenotype (*∗*: TSLPR expression is limited to gut DCs). Dendritic cells lacking HIF-1*α* under inflammatory conditions secrete steady high levels of IL-12 and thereby induce a robust activation of proinflammatory T cell populations. They upregulate transcripts of chemokines to attract more immune cells.

Fully in line with these findings that HIF-1*α* dampens the inflammatory response, but with a different consequence for the outcome of these mice, Flück et al. reported that HIF-1*α* is essential for dendritic cells to induce regulatory T cells (Tregs) in a model of acute DSS-colitis [48]. HIF-1*α* ^+f/+f^ mice and HIF-1*α* ^+f/+f^ CD11c-cre^wt/tg^ animals received equal amounts of DSS, but loss of dendritic HIF-1*α* caused more abundant inflammation and worse outcome for the animals. Mesenteric lymph nodes of HIF-1*α* ^+f/+f^ CD11c-cre^wt/tg^ animals showed significantly reduced expression of the anti-inflammatory cytokine IL-10 and of transforming growth factor- (TGF-) *β*—both are potent inducers of Tregs. In addition, the authors found a strongly reduced expression of aldehyde dehydrogenase (Aldh) 1a2, which is necessary for dendritic cells to catalyze retinal to retinoic acid (RA). RA also promotes Treg differentiation in the lymph node [7]. T cells of these lymph nodes in turn showed reduced expression of the retinoic acid receptor (RAR) *α* and diminished expression of the gut homing markers CCR9 and *α*4*β*7 integrin. HIF-1*α* deficiency in DCs thus abrogated their ability to induce Treg proliferation in secondary lymphoid organs and disrupted Treg homing towards the inflamed gut. Apart from the interplay between dendritic cells and T cells predominantly occurring in the mesenteric lymph nodes the authors could also show that the loss of HIF-1*α* by DCs affected the crosstalk between DCs and intestinal epithelial cells (IECs) causing IECs to produce more mucins and therefore overcome intestinal barrier damage. Another hint that loss of dendritic HIF-1*α* may affect the crosstalk between DCs and IECs was the finding that only HIF-1*α* ^+f/+f^ mice exhibited increased levels of thymic stromal lymphopoietin receptor (TSLPR) in DSS colitis. IECs release TSLP, which conditions mucosal DCs to noninflammatory tolerogenic DCs. A reduced expression of the respective receptor may drive DCs towards a more inflammatory phenotype.  Figure 1 summarizes the effects of (inflammatory) hypoxia and HIF-1*α* knockout on dendritic cells as it has been discussed above.

## 3. Conclusion

Dendritic cells normally have to function under conditions of inflammatory hypoxia. Not only inflammatory stimuli, but also hypoxia profoundly affects their cellular function. The key transcription factors regulating DCs' adaptation to conditions of (inflammatory) hypoxia are hypoxia-inducible factors (HIFs) 1 and 2.

Hypoxic culture of immature DCs induces expression of costimulatory molecules such as CD80, CD86, and HLA class II/MHCII [28, 29]. Whether these effects are dependent on hypoxia-inducible factor- (HIF-) 1 or not is still under debate [41, 42]. In contrast, HIF-1 is well recognized to affect migratory capacities of human and murine dendritic cells, most likely by shaping the cellular chemokine/chemokine receptor profile [21, 29]. Until now, only very few* in vivo* studies have addressed the role of HIFs for dendritic cell function; however these studies indicate that HIF-1 affects (i) differentiation of plasmacytoid dendritic cells in the bone marrow [45], (ii) migration of DCs towards secondary lymphoid organs [42], (iii) CD8^+^ T cell activation and release of pathogen-specific antibodies [46], (iv) expansion of CD8^+^ T cells and short-lived effector cells [47], and (v) induction of Tregs [48]. The studies of Hammami et al. [47] and Flück et al. [48] used the identical dendritic cell-specific HIF-1*α* knockout model (HIF-1*α* ^+f/+f^ CD11c-cre^wt/tg^) in different inflammatory settings. Very strikingly, both studies reported findings tending in the same direction but with very different outcomes for the treated animals. In both studies, dendritic cells lacking HIF-1*α* were the more potent inducers of an inflammatory response—with benefits for the outcome in* Leishmania* infection [47] but abundant inflammation and more severe illness in DSS colitis [48]. Clearly, more* in vivo* studies are needed to better understand the fragile balance between necessary induction of immunity to fight a disease and overactivation of immune cells damaging the host. Hypoxia-inducible factors may thereby be important transcriptional regulators in the balance of the immune status of dendritic cells.

## Figures and Tables

**Table 1:** Gene expression changes induced by hypoxia in human DCs.

Gene(s)	Changes in gene expression	Gene function	HIF dependency	References
*cd80*, *cd83*, *cd86*, *hla II*, and *cd40*	↑	Costimulatory molecules	cd83: yes [30] cd40: yes [32]	[28–30, 32]
*cx3cr1*, *ccr3*, *ccr2*, and *cxcr4*	↑	Chemokine receptors		[29]
*ccl13*, *ccl14*, *ccl18*, *ccl23*, *ccl24*, and *ccl26*	↓	Chemokines		[29]
*tnfsf14*	↑	Stimulation of T cells		[29]
*vegf*	↑	Angiogenesis	Yes	[29]
*il-8*, *mif*	↑	Inflammatory cytokines	il-8: dependency shown for human mesenchymal stem cells [39] mif: yes [40]	[29]
*trem-1*	↑	IRS receptor, triggers release of inflammatory cytokines	Yes	[36, 37]
*cxcl2*, *cxcl3*, *cxcl5*, *cxcl6*, and *cxcl8*	↑	Neutrophil recruitment	HRE found in cxcl2, cxcl5, and cxcl6	[34]
*ccl20*, *ccl3*, and *ccl5*	↑	Recruitment of activated T cells, monocytes, and immature DCs	HRE found in all of them	[34]
*ccl18*, *ccl23*	↓	Chemoattractants for naïve/resting T cells	Most likely indirect	[34]
*a2bar*	↑	Adenosine receptor	Yes	[35]

**Table 2:** Gene/protein expression changes induced by hypoxia in murine DCs.

Gene(s)/protein(s)	Changes in gene/protein expression	Gene/protein function	HIF dependency	References
*cd80*, *cd86*	Unaffected in differentiated BmDCs after 24 h of hypoxia ↑ after hypoxia + LPS ↑ after hypoxic differentiation of BmDCs	Costimulatory molecules	Yes No	[41] [42]
*nos2*	↑ after hypoxia + LPS	ROS production	Yes	[43]
IFN*α*4, IFN*α*12	↑ after hypoxia + LPS	Type I interferon	Most likely indirect	[44]
IFN-*β*	↑ after hypoxia + LPS	Type I interferon	Most likely indirect	[44]
IL-22	↑ after hypoxic differentiation of BmDCs	Inflammatory cytokine	Yes	[42]
CCR7	↑ after hypoxic differentiation of BmDCs	Chemokine receptor	Yes	[42]
*id2*	↑ after hypoxic differentiation of pDCs	Inhibits pDC lineage determination	Yes	[45]
